# Image sequence sorting algorithm for commercial tasks

**DOI:** 10.3389/frai.2024.1382566

**Published:** 2024-04-29

**Authors:** Guillaume Grelier, Miguel A. Casal, Alvaro Torrente-Patiño, Juan Romero

**Affiliations:** ^1^PhotoILike, Bergondo, Spain; ^2^RNASA Lab-IMEDIR, Department of Computer Science and Information Technologies, Faculty of Communication Science, University of A Coruña, A Coruña, Spain

**Keywords:** image ordering, semantic representation, image embedding, real estate, evolutionary computing, feature selection

## Abstract

**Introduction:**

The sorting of sequences of images is crucial for augmenting user engagement in various virtual commercial platforms, particularly within the real estate sector. A coherent sequence of images respecting room type categorization significantly enhances the intuitiveness and seamless navigation of potential customers through listings.

**Methods:**

This study methodically formalizes the challenge of image sequence sorting and expands its applicability by framing it as an ordering problem. The complexity lies in devising a universally applicable solution due to computational demands and impracticality of exhaustive searches for optimal sequencing. To tackle this, our proposed algorithm employs a shortest path methodology grounded in semantic similarity between images. Tailored specifically for the real estate sector, it evaluates diverse similarity metrics to efficiently arrange images. Additionally, we introduce a genetic algorithm to optimize the selection of semantic features considered by the algorithm, further enhancing its effectiveness.

**Results:**

Empirical evidence from our dataset demonstrates the efficacy of the proposed methodology. It successfully organizes images in an optimal sequence across 85% of the listings, showcasing its effectiveness in enhancing user experience in virtual commercial platforms, particularly in real estate.

**Conclusion:**

This study presents a novel approach to sorting sequences of images in virtual commercial platforms, particularly beneficial for the real estate sector. The proposed algorithm effectively enhances user engagement by providing more intuitive and visually coherent image arrangements.

## 1 Introduction

In the digital age, the potency of e-commerce hinges significantly on the art of presentation, especially within the visual-centric realm of online marketing. A paramount aspect of this presentation is the strategic deployment of images to convey accurate and appealing product narratives. This strategy, however, faces its quintessential challenge in ensuring the transmission of precise information (Jin and Gallimore, 2010). The conventional reliance on detailed written descriptions is undeniably foundational, yet the transformative power of images in enhancing these descriptions cannot be overstated. Quality imagery has a critical role in capturing attention and bolstering consumer confidence, ultimately driving purchase intentions (Chen and Teng, 2013; Di et al., 2014; Zakrewsky et al., 2016).

This visual imperative is particularly pronounced in the realm of real estate marketing. Here, the breadth and quality of property images directly influence potential buyers' interest (Thaler and Koch, 2023), and distinguish listings in a crowded market (Siebert and Seiler, 2022). The crux of effective visual presentation, therefore, lies in the intuitive and logical sequencing of these images (David Koch and Mayr, 2021). A coherent arrangement that clusters images by room or similarity not only eases navigation but significantly reduces cognitive strain, thereby enhancing the overall decision-making process (Cacioppo et al., 1996; Garbarino and Edell, 1997; Franco-Watkins et al., 2006).

Yet, the rapidly evolving real estate sector, with its increasing tilt toward automation in listing processes, often overlooks the nuanced task of image sequencing. The indiscriminate uploading of property photos without regard to their order poses a glaring challenge, highlighting a gap in the automated sorting mechanisms explored in other domains (Chaudhuri et al., 2018; Fan et al., 2022). The pivotal question, then, revolves around developing an algorithm capable of organizing these images in a manner that respects the inherent grouping without prior knowledge of these groups' specifics.

This problem can be seen and formalized as an Ordering Problem (see Section 2.1). While one approach might involve using a clustering algorithm to generate potential sub-groups, ordering elements based on these sub-groups can be challenging. If the number of clusters obtained from the algorithm differs from the initial number of subsets, achieving an accurate final order becomes unlikely. Therefore, this paper suggests an alternative method that increases the likelihood of obtaining an accurate order without requiring prior knowledge of the number of clusters. Note that the challenge arises precisely from not knowing the exact number of distinct rooms present; otherwise, applying a clustering algorithm would suffice.

Then, addressing the ordering problem requires a foundational shift toward a methodology grounded in the principle of similarity. By devising a similarity metric among the images, we aim at defining an algorithm solution to organize the elements in a way that those belonging to the same subset (e.g., images of the same room) are placed adjacently, thereby preserving the partition's integrity. This approach, while methodical, navigates the complexity of limited knowledge about the partition's structure and the count of subsets, challenging the straightforward application of clustering algorithms.

## 2 Methods

### 2.1 Ordering problem

An ordering problem consists of finding a permutation that maximizes (or minimizes) a certain quantity, and it can be mathematically formalized in a general manner, focusing on identifying permutations of elements in set A that preserve the structure of a given partition P. In simpler terms, the objective is to find orders of elements in A such that elements belonging to each subset within partition P appear consecutively (see **Definition 2.2**). To illustrate, consider A as a collection of bedroom photos, with P being a partition where each subset comprises photos of the same bedroom.

For notation purposes, let *n* ≥ 1 be a finite set, and *S*_*n*_ denote the symmetric group of order *n*. The cardinality of a set *A* is denoted by |*A*|. Any σ ∈ *S*_*n*_ is referred to as a permutation. The vector space of real square matrices of size *n* is denoted by $M_{n}\left(\mathbb{R}\right)$.

The ordering problem can be seen as a variation on the traveling salesman problem, where the aim is to travel through all the towns once and only once, minimizing the total distance and without returning to the starting point. Formally, the definition is the following:

Definition 2.1. Let $M={\left(m_{i,j}\right)}_{1\leq i,j\leq n}\in M_{n}\left(\mathbb{R}\right)$ and *S* ⊂ *S*_*n*_. The **ordering problem associated to** ***M*** **and** ***S*** is the following optimization problem:


$$\underset{\sigma \in S}{\max}\overset{n-1}{\underset{i=1}{\sum }}m_{\sigma \left(i\right),\sigma \left(i+1\right)}.$$

For simplicity, we define the function *f*_*M*_:*S*_*n*_ → ℝ by $f_{M}\left(\sigma \right)=\overset{n-1}{\underset{i=1}{\sum }}m_{\sigma \left(i\right),\sigma \left(i+1\right)}$. The ordering problem associated to *M* and *S* consists in maximizing *f*_*M*_ over *S*.

Of course, since the set over which we are minimizing is finite, there is always a solution. The difficulty lies in finding a solution computationally in a reasonable time. In fact, it can be proved that the previous problem is an NP-hard problem in combinatorial optimization when *S* = *S*_*n*_ (Opatrny, 1979).

There are many well-known algorithmic methods for solving this kind of problem. If *n* is small, it is possible to go through all the permutations of *S*_*n*_ in order to maximize *f*_*M*_. However, if *n* is large (typically, when *n* > 10), the cardinal of *S*_*n*_ being *n*! we need to find an alternative method. There are a number of methods for finding an approximate solution to the traveling salesman problem. Here we will quickly present a well-known algorithm that is easy to implement, fast and more than sufficient for our purposes: the nearest neighbor algorithm. Depending on the problem, it may of course be necessary to use a more complex algorithm to maximize *f*_*M*_ since, in general, the nearest neighbor algorithm does not give an optimal solution.

Let $M={\left(s\left(x_{i},x_{j}\right)\right)}_{1\leq i,j\leq n}\in M_{n}\left(\mathbb{R}\right)$. Define *f*_*M*_ as in **Definition 2.1**. Let *x*_*i*_1__ be an aleatory starting point. We then choose the element *x*_*i*_2__ closest to it, i.e., the one that maximizes the quantity *s*(*x*_*i*_1__, *x*_*i*_2__). We repeat the process, looking for the element *x*_*i*_3__ closest to *x*_*i*_2__. When all the elements have been used, we stop. For each starting point *x*_*i*_, we obtain a permutation σ_*i*_. The final permutation is obtained by choosing the σ_*i*_ permutation satisfying $f_{M}\left(\sigma_{i}\right)=\underset{1\leq j\leq n}{\max}f_{M}\left(\sigma_{j}\right)$. The pseudo-code detailed in Algorithm 1:

**Algorithm 1:**
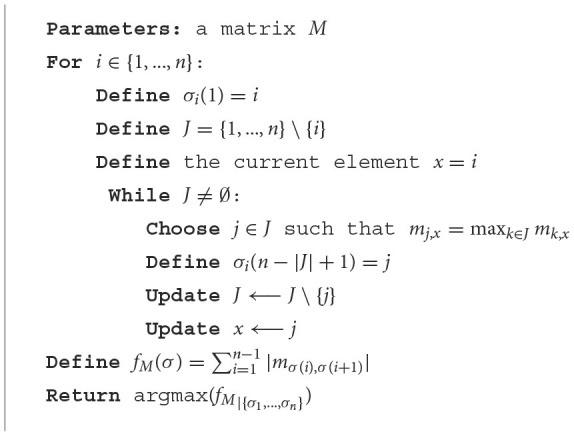
Nearest neighbor algorithm.

In many applications, a starting point can be necessary. For example, in our case, it might be interesting to choose the photo with the highest commercial appeal as the initial point. If a starting point *x*_*i*_0__ is required, the for loop should of course be removed and only the permutations σ such that *x*_σ(1)_ = *x*_*i*_0__ are considered.

### 2.2 Ordering algorithm with unknown partition

Definition 2.2. Let *n* ≥ 1 and let $A=\left\{x_{1},...,x_{n}\right\}$ be a set of cardinal *n*. Let $P=\left\{A_{1},...,A_{p}\right\}$ be a partition of A. We say that a permutation σ ∈ *S*_*n*_ **preserves** P if


$$\forall i\in \left\{1,...,p\right\}\;\exists j\in \left\{1,...,n\right\}\;\text{such that }x_{\sigma \left(j\right)},x_{\sigma \left(j+1\right)},...,x_{\sigma \left(j+|A_{i}|-1\right)}\in A_{i},$$

i.e., for all *i* ∈ {1, ..., *p*}, the elements of *A*_*i*_ are found successively in the ordered sequence *x*_σ(1)_, *x*_σ(2)_, ..., *x*_σ(*n*)_. The set of permutations preserving P will be denoted by O(P).

The partition P is not known, and we want to find a method to discover a permutation that preserves P. We will see that such permutations can be obtained by solving an ordering problem associated with a well-chosen matrix *M*.

In our case, O(P) represents the set of permutations in which the photos belonging to the same room are consecutive. Since the objective is to determine the permutations that preserve P, it is useful to determine how many there are.

Proposition 2.3. Let A be a finite set and let $P=\left\{A_{1},...,A_{p}\right\}$ be a partition of A. Then $|O\left(P\right)|=p!\prod_{k=1}^{p}|A_{k}|!$.

Proof.There are *p*! ways of ordering the sets *A*_*i*_. Then, in each set *A*_*i*_, there are |*A*_*i*_|! ways of permuting its elements. The result is then obtained.

Although intuitively trivial, we deduce that any permutation preserves P if and only if *p* = 1 or *p* = *n*. This corresponds to cases where the partition has only one set, or where all the sets in the partition are singletons.

Since P is not necessarily known, we have to find a way to determine the set of permutations preserving P. One way of doing this is to determine the functions defined on *S*_*n*_ whose maxima are permutations that preserve P:

Definition 2.4. Let *f*:*S*_*n*_ → ℝ. We say that *f* is **compatible with** P if argmax(f)⊂O(P) and we write f∈C(P). If moreover, *f* ≥ 0, we write $f\in C^{+}\left(P\right)$.

It is clear that there always exists a function *f* which is compatible with P. In fact, let Γ⊂O(P) and define *f* by


$$f(\sigma )=\left\{ \begin{array}{l} 1\text{ if }\sigma \in \Gamma \\ 0\text{ if not} \end{array}\right.$$

It is clear that f∈C(P). The following result shows that there are many functions compatible with P. We recall that $C\left(S_{n}\right)$ is the set of continuous real functions defined on *S*_*n*_ (since *S*_*n*_ is discrete, this refers to simply real functions defined on *S*_*n*_).

Proposition 2.5. $C\left(S_{n}\right)=C^{+}\left(P\right)-C^{+}\left(P\right)$.

Proof. Let $f\in C\left(S_{n}\right)$ such that *f* ≠ 0. Since $C^{+}\left(P\right)$ is stable by adding a positive constant, we can and do suppose that *f* > 0. We define $f_{1},f_{2}\in C\left(S_{n}\right)$ by


$$f_{1}(\sigma )=\{\begin{array}{ll} f(\sigma ) & \text{if }\sigma \in O(P)\\ 0 & \text{if not} \end{array}\text{ and }f_{2}(\sigma )=\{\begin{array}{ll} 0 & \text{if }\sigma \in O(P)\\ -f(\sigma ) & \text{if not} \end{array}$$

It is clear that *f* = *f*_1_ − *f*_2_. Since *f* > 0, *f*_1_ and *f*_2_ reached their maxima on O(P), i.e., $f_{1},f_{2}\in C\left(P\right)$. Now pick α > 0 such that *g*_1_: = *f*_1_ + α > 0 and *g*_2_: = *f*_2_ + α > 0. We have $f=g_{1}-g_{2}\in C^{+}\left(P\right)-C^{+}\left(P\right)$.

If P is unknown, it can therefore be difficult to determine a function *f* which is compatible with P. Depending on the desired order, the *f* function must be chosen on the basis of the data so that it is compatible with P. Then, by maximizing *f*, we will obtain an acceptable solution associated to P.

We are going to look for functions that are compatible with P and have a particular form:

Definition 2.6. Let $M={\left(m_{i,j}\right)}_{1\leq i,j\leq n}\in M_{n}\left(\mathbb{R}\right)$. The permutations maximising the function *f*_*M*_:*S*_*n*_ → ℝ defined by


$$f_{M}\left(\sigma \right)=\overset{n-1}{\underset{i=1}{\sum }}m_{\sigma \left(i\right),\sigma \left(i+1\right)}$$

are called **longest paths of** **M**. We say that *M* is **compatible with** P if *f*_*M*_ is compatible with P. In other words, *M* is compatible with P if any of its longest paths is a permutation preserving P.

One can ask if any function of C(P) is of the form *f*_*M*_ for some $M\in M_{n}\left(\mathbb{R}\right)$. Unfortunately it is easily seen that ${\left\{f_{M}\right\}}_{M\in M_{n}\left(\mathbb{R}\right)}$ is a strict subset of C(P). In fact, the map $T:M_{n}\left(\mathbb{R}\right)\to C\left(S_{n}\right)$ defined by *T*(*M*) = *f*_*M*_ is linear. It follows that ${\left\{f_{M}\right\}}_{M\in M_{n}\left(\mathbb{R}\right)}\subset \text{Im}\left(T\right)$ with dim(Im(*T*)) ≤ *n*^2^. We have that dim(span(C(P)))=n! by Proposition 2.5. In particular, if *n* > 1, we have that ${\left\{f_{M}\right\}}_{M\in M_{n}\left(\mathbb{R}\right)}⊊C\left(P\right)$. Although the *f*_*M*_ functions form a small subset of C(P), they are the functions that are most natural to define in practice. In fact, the matrix *M* can be seen as a similarity matrix where *m*_*i, j*_ is the similarity coefficient between *x*_*i*_ and *x*_*j*_.

Definition 2.7. Let $s:A^{2}\to \mathbb{R}$ and define *M* = (*s*(_*x*_*i*_, *x*_*j*_))1 ≤ *i, j* ≤ *n*_. We say that *s* **describes** P if *M* is compatible with P.

The *best* function describing P is defined as follows:

Example 2.8. Let $s:A^{2}\to \mathbb{R}$ defined by *s*(*x*_*i*_, *x*_*j*_) = 1 if there exists *k* ∈ {1, ..., *p*} such that *i, j* ∈ *A*_*k*_ and *s*(*x*_*i*_, *x*_*j*_) = 0 if not. Define *M* = (*s*(_*x*_*i*_, *x*_*j*_))1 ≤ *i, j* ≤ *n*_. It is easy to see that *f*_*M*_(σ) ≤ *n*−*p* for all σ ∈ *S*_*n*_ with equality if and only if σ∈O(P). In particular, *M* is compatible with P, i.e., *s* describes P.

We are now ready to propose a simple algorithm (Algorithm 2) to find permutations that are compatible with a given partition:

**Algorithm 2:**
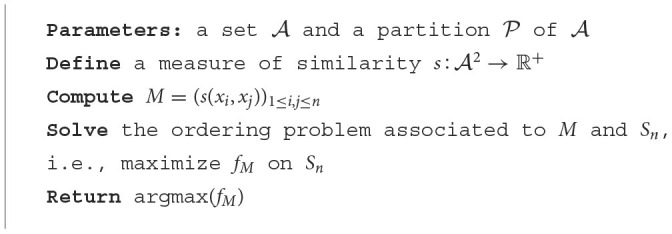
Ordering algorithm.

The entire challenge lies in finding a similarity measure that captures the essence of P. In our case, the goal is to discover a similarity measure where the value is high for photos of the same room and low for photos of different rooms.

It is possible that the longest path does not pass through two points with high similarity. This phenomenon is amplified in practice when a starting point is chosen. Consider the similarity matrix


$$M={\left(s(x_{i},x_{j})\right)}_{1\leq i,j\leq 4}=\left(\begin{array}{llll} 1 & 0.5 & 0.7 & 0.5\\ 0.5 & 1 & 0.5 & 0.2\\ 0.7 & 0.5 & 1 & 0.5\\ 0.5 & 0.2 & 0.5 & 1 \end{array}\right).$$

Suppose we want our starting point to be *x*_1_. In view of the similarity matrix, points *x*_1_ and *x*_3_ are the most similar. We therefore expect them to be part of the same group and the optimal path to visit them successively. However, the maximum path starting at *x*_1_ is given by *x*_1_, *x*_2_, *x*_3_, *x*_4_ and has a length of 1.5, while the path *x*_1_, *x*_3_, *x*_2_, *x*_4_ has a length of 1.4. It can therefore be useful to force the algorithm to pass successively through pairs of points with a high similarity. For this reason, we propose the following modification (Algorithm 3) to the previous algorithm that can be applied to any matrix *M*.

**Algorithm 3:**
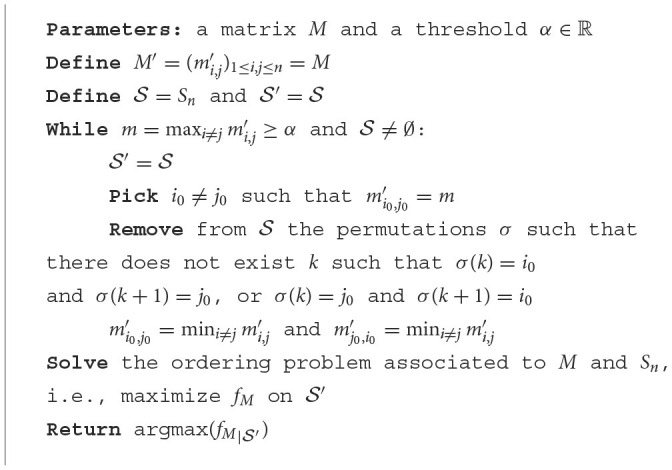
Improved ordering algorithm.

The previous algorithm forces pairs of elements whose similarity is sufficiently high to follow each other in the final order. Of course, if a starting point *x*_*i*_0__ is desired, then S should be defined as the subset of *S*_*n*_ consisting of the permutations σ satisfying *x*_σ(1)_ = *x*_*i*_0__. It should be borne in mind that the use of a starting point, although necessary for certain applications, can only worsen the performance of the algorithm.

### 2.3 Dataset

**Figure 1:**
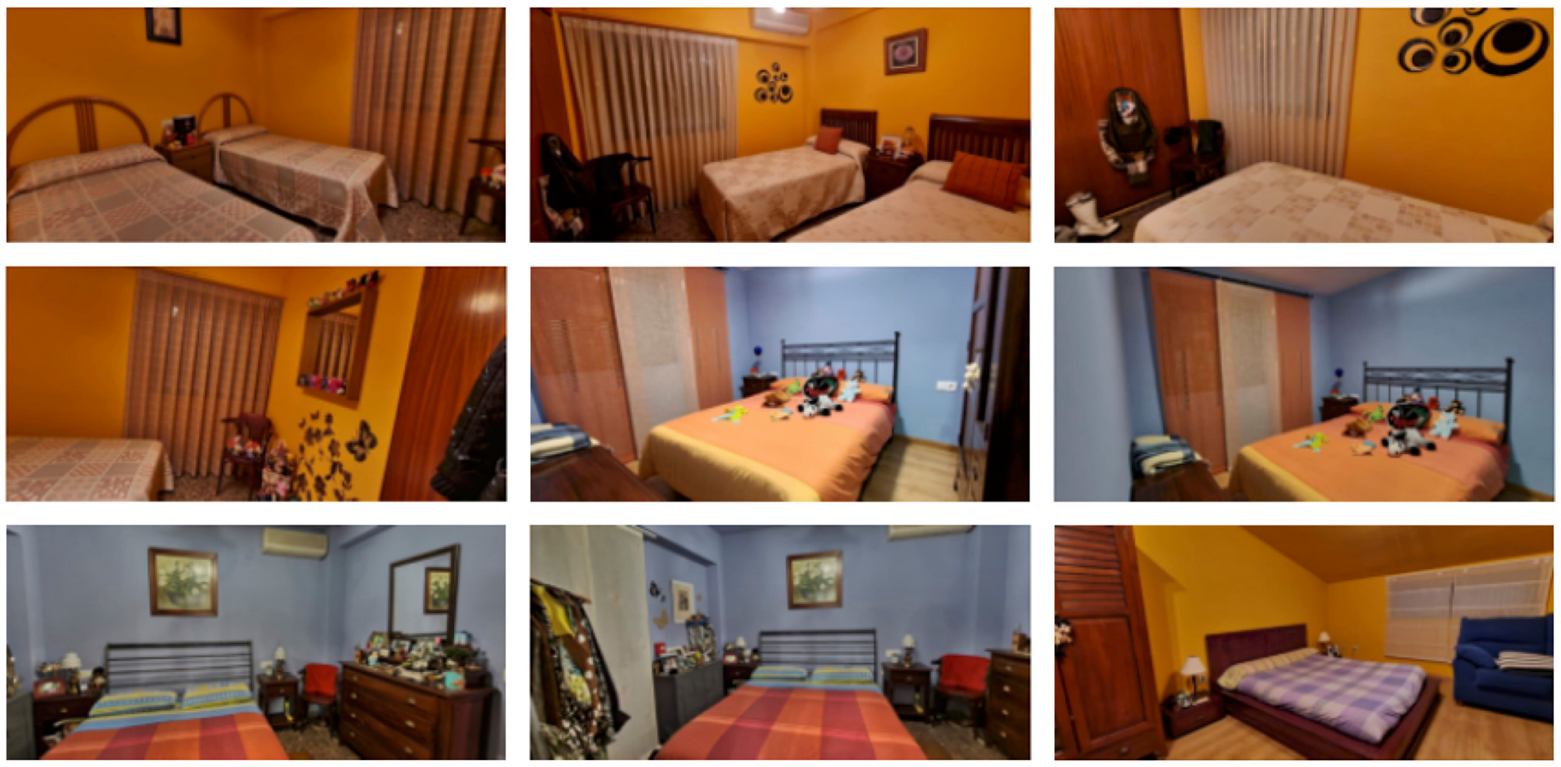
Photos of bedrooms in an ad.

Our dataset comprises 295 property ads, each containing varying numbers of photos. Each photo is labeled, identifying the specific room category it belongs to (e.g., bedroom, living room, exterior,...). For the purpose of this analysis, we focus solely on bedroom photos within each property listing, since it is likely to find a varying number of bedrooms across ads. Therefore, our dataset can be represented as a collection of sets: $C={\left\{A_{i}\right\}}_{1\leq i\leq 295}$, where each $A_{i}$ corresponds to a set of *n*_*i*_ bedroom photos (with potential errors from the initial model, although this doesn't impact the ordering algorithm's structure). For each $A_{i}$, the objective is to arrange the photos in such a manner that pictures of the same bedroom are contiguous. Consequently, for each $A_{i}$, there exists an unknown partition $P_{i}$ of $A_{i}$, where each subset comprises photos of the same bedroom. An example of bedroom-categorized advertisement photos is shown in Figure 1.

In case $|P_{i}|=1$ or $|P_{i}|=n_{i}$, any order would be a solution (see Proposition 0.0.3). We will therefore eliminate the elements $A_{i}$ verifying this condition since, regardless of the model, the result will be good. After these eliminations, our dataset C contains 211 elements. In order to avoid over-complicating the notations, we will still write $C={\left\{A_{i}\right\}}_{1\leq i\leq k}$ with *k* = 211 and where each $A_{i}$ is a set of *n*_*i*_.

### 2.4 Application to image ordering

Recall that our dataset a set of $C={\left\{A_{i}\right\}}_{1\leq i\leq k}$, where *k* = 211 and each $A_{i}$ is a set of *n*_*i*_ bedroom photos. For each *A*_*i*_, the objective is to order the photos in such a way that the photos of the same room follow one another. That is, if $P_{i}$ is the unknown partition of *A*_*i*_ where each set of which consists of photos of the same room, we want to find a permutation preserving $P_{i}$.

#### 2.4.1 Embedding vectors

An embedding vector serves as a condensed representation that encapsulates the informational essence held within an image or text (Gutiérrez and Keith, 2019; Schwalbe, 2022). Through a complex process of feature extraction and transformation, an image is translated into a numerical vector that captures key visual characteristics and patterns. This vector, known as the embedding vector, effectively distills the image's relevant information into a more compact and manageable format. This process enables efficient storage, comparison, and analysis of images, making it an indispensable tool in various applications such as image retrieval, similarity assessment, and even machine learning tasks where image data is a crucial input.

This embedding process is commonly achieved through the utilization of a neural network known as an image encoder. The image encoder is specifically designed to take raw image data as input and transform it layer by layer into a meaningful and compact embedding vector. This network leverages its learned weights and architecture to extract hierarchical features from the image, gradually abstracting away from low-level pixel information to higher-level semantic representations. As the image progresses through the layers of the encoder, its distinctive features and attributes are progressively distilled into the final embedding vector, creating a powerful and condensed representation that captures the image's essence. This integration of deep learning techniques within the image embedding process enhances the ability to capture intricate patterns, allowing for more effective downstream tasks that rely on these embeddings.

Notation 2.9. When using an image encoder *e*, the dimension of the embedding vector will be denoted as *m*. Moreover, if *I* is an image, the corresponding embedding vector will be denoted by *e*(*I*) ∈ ℝ^*m*^.

In our case, we will use the image encoders of two algorithms: CLIP (Radford et al., 2021) and BLIP (Li et al., 2022), two of the currently most effective algorithms.

##### 2.4.1.1 CLIP

The embedding vector obtained thanks to the image encoders of the CLIP algorithm is a vector of a vector of *m* = 512 coordinates. To obtain this image encoding, CLIP uses a deep neural network called the “image encoding network”. This network is a crucial part of the overall CLIP model, which combines image encoding with text encoding to perform multimodal tasks. The image encoding network is pretrained on a large dataset of images from the internet in an unsupervised manner before being fine-tuned using the contrastive process, as mentioned in the previous response. Through this training, the network learns to extract important and semantically meaningful visual features from images, creating a 512-dimensional vector that captures key information about the image.

Notation 2.10. The image encoder of CLIP will be denoted by *c*.

##### 2.4.1.2 BLIP

The BLIP image encoder returns an embedding matrix of dimensions 32 × 256 (meaning that *m* = 8192). They incorporate a visual transformer as our image encoder, which breaks down input images into patches and converts them into a sequence of embeddings. An additional token is included to represent the overall image feature. This choice offers advantages over the use of pretrained object detectors for visual feature extraction, as it is computationally more efficient. This approach has gained popularity in recent methodologies for its effectiveness in image processing tasks.

Notation 2.11. The image encoder of BLIP will be denoted by *b*.

#### 2.4.2 Image ordering algorithm

In order to compare two embedding vectors, we will consider a similarity measure *S*:(ℝ^*m*^)^2^ → ℝ^+^ and then calculate the arithmetic mean of this measure evaluated on the images themselves and on their symmetrical images. We then obtain a similarity measure *s* which will be used to order the images. Its calculation is given in the following algorithm. Before that, we just introduce the following notation:

Notation 2.12. Let *m* ≥ 1. If *x* ∈ ℝ^*m*^ and *v* ∈ ℝ^*m*^ is a vector such that *v*_*i*_ ∈ {0, 1} for all *i* ∈ {1, ..., *m*}, we denote by *x*_*v*_ the vector given by $v_{x}=\overset{m}{\underset{i=1}{\sum }}v_{i}x_{i}e_{i}$ where (*e*_*i*_)_1 ≤ *i* ≤ *m*_ is the canonical basis of ℝ^*m*^.

Many image encoders are sensitive to axial symmetries due to the nature of the neural networks it employs. The deep neural networks are designed to learn and recognize patterns and features in images. When the image is transformed by axial symmetry (horizontal or vertical flipping), the spatial arrangement of visual elements changes. Consequently, the network may extract different features from the transformed image compared to the original one. Since these algorithms encode images based on these extracted features, the resulting feature vector representing the transformed image will differ from the one representing the original image. As a result, when these algorithms compare an image with its axially symmetrical counterpart or with textual descriptions, the differences in feature extraction can influence the perceived similarity between the images or the alignment with the provided text. This is why we propose the following computation (Algorithm 4) of the similarity matrix:

**Algorithm 4:**
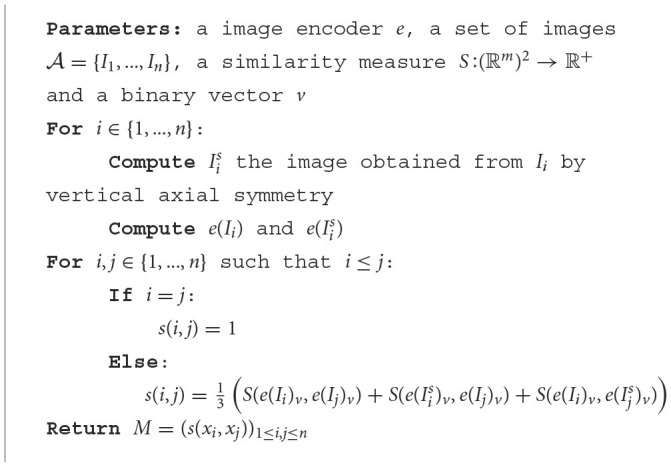
Similarity matrix.

The *v* vector is a binary vector which selects variables. If there is no knowledge of the importance of the variables, it is sufficient to consider that *v* is the vector of ℝ^*m*^ whose coordinates are all 1. We will then propose a method for selecting the important variables using a genetic algorithm (see Section 2.4.3).

The choice of the similarity measure *S* depends, of course, on the problem in question. For example, one can consider the cosine similarity or metrics based on geometrical distances (Martinez et al., 2024) given by $S\left(x,y\right)=\frac{1}{1+||x-y||}$ where ||.|| is a norm of ℝ^*m*^. Here we will use the ℓ_2_-norm ||.||_2_. In our case, the measure that gave the most significant results is the cosine similarity.

We are now ready to present our image ordering algorithm (Algorithm 5) as follows:

**Algorithm 5:**
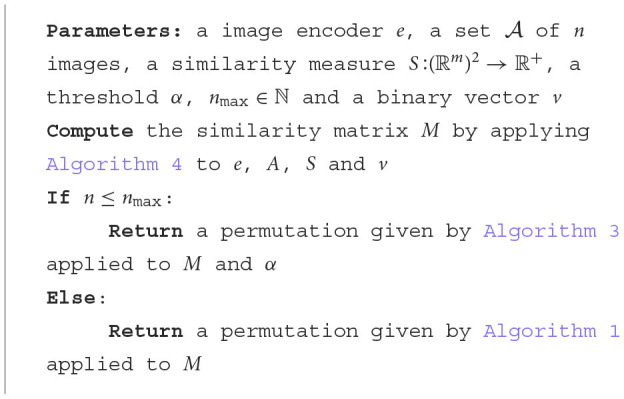
Ordering algorithm.

#### 2.4.3 Feature selection using genetic algorithm

Feature selection utilizing a genetic algorithm is pivotal for refining our understanding and application of multivariable analysis in this study. Specifically, we delve into the intricate relationships between multiple predictor variables—features extracted from image embeddings—and the response variable, which is the accuracy of image sequencing, quantified as the well-ordering rate. Through this analysis, we aim to uncover the most impactful features that enhance the precision of image ordering and explore the dynamics of their interactions.

In our study, multivariable analysis critically examines the interplay between multiple predictor variables, specifically features extracted from image embeddings, and the response variable—image sequencing accuracy, measured by the well-ordering rate. This analysis is pivotal for identifying features that significantly enhance ordering accuracy and understanding their synergistic interactions.

In this endeavor, the genetic algorithm emerges as a cornerstone, facilitating the discernment of a subset of features within the high-dimensional embedding vectors that are paramount for efficacious image sorting. This entails a meticulous process of evaluating the performance imparted by various feature subsets on the image ordering task, followed by an iterative refinement of these subsets to optimize the well-ordering rate.

Feature selection is not merely about enhancing algorithmic accuracy; it's also about augmenting data clarity by mitigating noise and bolstering computational efficiency. Our goal is to pinpoint the specific coordinates within the embedding vector that hold significance for image classification. With a preliminary understanding that the binary vector *v* constituted all ones, the introduction of a genetic algorithm to refine the selection of *v* represents a strategic pivot. We focus on the embedding vectors generated by BLIP, specifically targeting the pivotal coordinates for image ordering. Given that these vectors comprise 32 sub-vectors, each with 256 coordinates, our selection process simplifies each sub-vector into a singular variable for feature selection.

**Figure 2:**
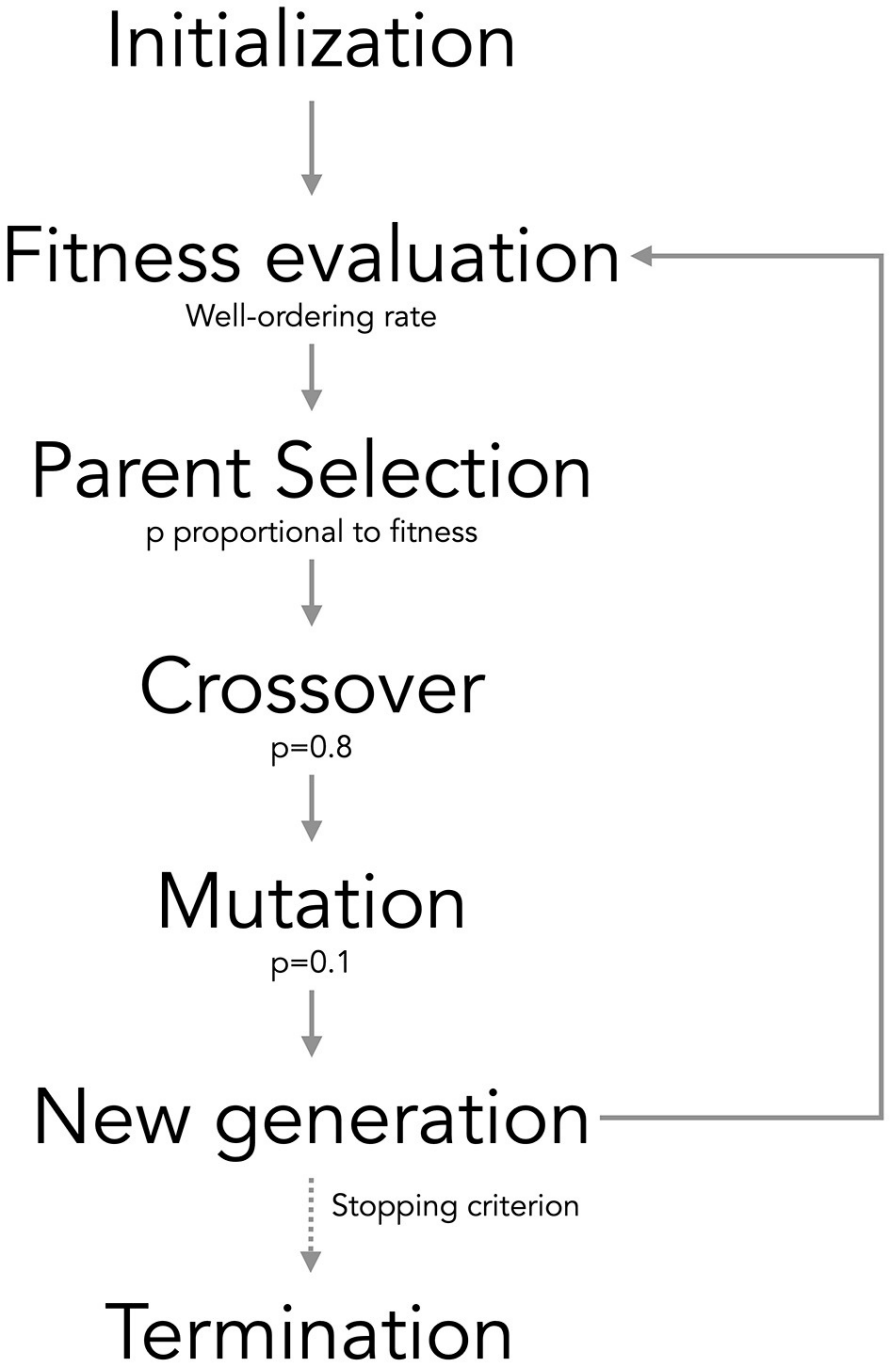
Scheme of the genetic algorithm used.

Genetic algorithms, a well-entrenched optimization technique (Slowik and Kwasnicka, 2020; Sohail, 2023), are adeptly employed for variable selection. The aim is to isolate an optimal subset of variables that elevate the designated fitness function. Within the population of the genetic algorithm, each individual signifies a potential subset of variables, denoted by a binary vector that marks the inclusion (1) or exclusion (0) of each variable (each sub-vector). This systematic approach culminates in the identification of the optimal variable subset, significantly enhancing the efficiency and efficacy of image ordering in our dataset. The primary steps of the genetic algorithm are illustrated in Figure 2, with detailed explanations provided below:

(1) Initialize population: The GA initiates by creating a population of individuals, each possessing a binary string of 0s and 1s, indicating the inclusion (1) or exclusion (0) of variables in the subset.
(2) Fitness evaluation: Compute the fitness score for each individual in the population. In this specific application, the fitness function is defined as the well-ordering rate, where a higher fitness score signifies a more effective subset of variables.
(3) Parent selection: Individuals are chosen for reproduction based on their fitness scores. The probability of selecting each individual as a parent is proportional to its fitness score such that $p_{\text{selection}}=\frac{f_{i}}{\underset{i}{\sum }f_{i}}$.
(4) Crossover: Crossover is performed with a probability (*p*_crossover_ = 0.8). Two parents are randomly selected from the parent pool, and a crossover point is chosen. The offspring are generated by swapping the binary strings of the parents at the crossover point, creating two new individuals.
(5) Mutation: Mutation occurs with a specified probability (*p*_mutation_ = 0.1) for each bit of an individual. If mutation occurs, the bit is flipped (0 becomes 1, and vice versa), introducing exploration and aiding the algorithm in escaping local optima.
(6) Next generation: The new population is formed by repeating the crossover and mutation process for the entire population.
(7) Termination: The GA runs for a predefined number of generations. Upon completion of the specified number of generations, the algorithm returns the best individual found during the process, representing the optimal subset of variables.

As aforementioned, the fitness function is given by the well-ordering rate, that is detailed in Algorithm 6:

**Algorithm 6:**
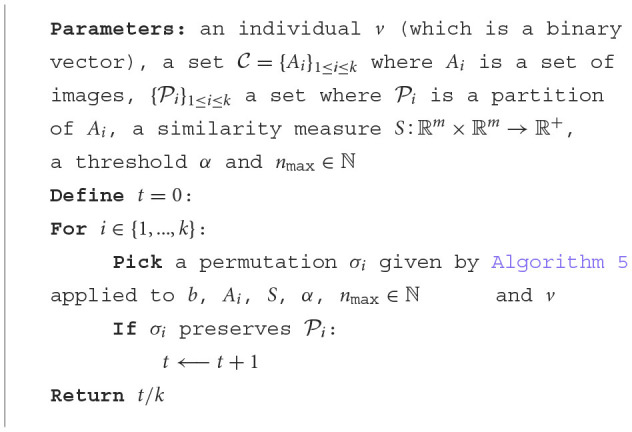
Fitness function.

## 3 Results

In this section, we present the results of Algorithm 5 applied to our problem. To do so, we will evaluate the results based on the numbers of adds that are well-ordered. This means, with an accuracy metric for all the ads called the well-ordering rate:

Definition 3.1. Let $C={\left\{A_{i}\right\}}_{1\leq i\leq k}$ our set of sets of images and for each *i* ∈ {1, ..., *k*}, let $P_{i}$ be a partition of $A_{i}$ and σ_*i*_ ∈ *S*_*n*_*i*__ (where *n*_*i*_ = |*A*_*i*_|). We define **well-ordering rate** by


$$\tau =\frac{1}{k}|\left\{i\in \left\{1,...,k\right\}\;:\;\sigma_{i}\;\text{preserves }P_{i}\right\}|.$$

Here $A_{i}$ is a set of images, $P_{i}$ is the partition of $A_{i}$ whose sets contains the photos of the same bedroom and σ_*i*_ is a permutation obtained thanks to Algorithm 5. Results are shown in Table 1.

**Table 1:** Results with symmetrization.

**Encoder *e***	***n*_max_**	**α**	***S*(*x, y*)**	**τ**
*c*	10	0.9	$\frac{\langle x,y\rangle }{||x|{|}_{2}||y|{|}_{2}}$	69.7
*c*	10	0.9	$\frac{1}{1+||x-y|{|}_{2}}$	66.4
*c*	0	–	$\frac{\langle x,y\rangle }{||x|{|}_{2}||y|{|}_{2}}$	65.4
*c*	10	0.9	$\frac{1}{1+||x-y|{|}_{2}}$	66.4
*b*	10	0.9	$\frac{\langle x,y\rangle }{||x|{|}_{2}||y|{|}_{2}}$	81

It is worth recalling that the value of *n*_max_ determines the threshold at which we employ an approximate solving method to determine an optimal solution. The more you increase *n*_max_, the better the results will be. However, for computational reasons, it is wise to choose a value between 10 and 12.

As mentioned in Section 2.4.2, image encoders are sensitive to axial symmetries. We can indeed confirm this here. Results from replacing the similarity measure of Algorithm 4 by *s*(*i, j*) = *S*(*e*(_*I*_*i*_)*v*_, *e*(_*I*_*j*_)*v*_) are shown in Table 2.

**Table 2:** Results without symmetrization.

**Encoder *e***	***n*_max_**	**α**	***S*(*x, y*)**	**τ**
*c*	10	0.9	$\frac{\langle x,y\rangle }{||x|{|}_{2}||y|{|}_{2}}$	66.4
*b*	10	0.9	$\frac{1}{1+||x-y|{|}_{2}}$	80.6

One thing that is interesting to note is that, unlike CLIP, BLIP seems to have little sensitivity to image orientation, since the results are very similar (with τ varying by only 0.4% for BLIP, compared to 3.3% for CLIP.)

Previous results were obtained without performing any feature selection. We then upgraded the model by applying a genetic algorithm to perform feature selection (see Section 2.4.3). Note that since the results are significantly better with BLIP than with CLIP, we confine ourselves here to improving on the results of the algorithm using BLIP. We used cosine similarity for the fitness similarity measure *S*. Considering a population of 50 and 10 generations, we obtained a vector *v*_GA_ that we can use in Algorithm 5. For computational reasons, we lower *n*_max_ to 9 and we obtained that well-ordering rate has therefore increased by 4.8%, as shown in Table 3.

**Table 3:** Results with GA.

**Encoder *e***	***n*_max_**	**α**	***S*(*x, y*)**	**τ**
*b*	9	0.9	$\frac{\langle x,y\rangle }{||x|{|}_{2}||y|{|}_{2}}$	85.8

## 4 Discussion

In the discussion of our findings, it's important to contextualize the implications of our approach for the broader field of image processing and its applications in online commerce. Our method, while specifically tailored to address the challenge of sorting property listing images, holds potential for a wide range of applications where image order and selection are critical. By grounding our solution in a general mathematical framework and leveraging semantic image embedding, we offer a path forward for similar challenges across various domains.

The employment of a genetic algorithm for semantic variable selection underscores the adaptability of our approach. This adaptability is crucial, as it suggests that with appropriate tuning, our method can be extended to other types of datasets or sorting criteria. For instance, in e-commerce, where the visual presentation of products can significantly influence consumer behavior, our algorithm could be used to optimize the arrangement of product images to highlight features most likely to appeal to customers.

Irrespective of the specific field of application, the predominant methodology for image sorting has traditionally leveraged clustering techniques, which range from classical machine learning approaches like *k*-means or *k*-nearest neighbors to more advanced neural network frameworks (Rorissa and Hastings, 2004; Liang et al., 2023). While such methods offer rapid processing and effectively categorize images into distinct subsets, they fundamentally execute a classification-centric sorting strategy. This approach does not account for the ordering within these subsets, nor does it address the aspect of sequentiality that is critical in the context of the current study and others focused on e-commerce applications.

Previous research has explored the automation of product image sequencing in the e-commerce domain (Chaudhuri et al., 2018; Fan et al., 2022). However, the realm of real estate listings presents a notably more intricate challenge. This complexity stems from the diversity and uncertainty associated with subproducts, such as the various rooms and types of rooms, each with their unique 3D shapes and elements. This scenario is markedly different from simpler products like smartphones or clothing, where the variability and spatial considerations are significantly less pronounced. Consequently, the methodologies and solutions developed for simpler product categories fall short of addressing the intricate demands and nuances of the real estate sector.

One of the notable challenges in our study is associated with the methodology grounded in similarity distance metrics. This approach inherently positions images with high similarity—including identical ones—next to each other in the sequence, inadvertently overlooking the potential issue of redundancy. Our research operates under the presumption that each image contributes a meaningful and complementary information when compared to others. However, this assumption might not always hold true, particularly in scenarios where photographs are captured and selected for marketing purposes, with the aim of automating the process of compiling images for a commercial advertisement. An example of redundancy within the realm of real estate marketing might involve multiple shots of the same room captured from slightly varied angles, which, in essence, fail to furnish additional valuable information to the prospective buyer.

In practical applications, especially in digital marketing and online advertising, the presence of redundant images could dilute the effectiveness of the visual narrative, potentially overwhelming or disengaging the target audience. Recognizing and eliminating redundancy not only streamlines the content but also enhances the user experience by presenting a concise and focused array of images that collectively convey a more powerful message. Image sequence generation for products in e-commerce has been already addressed taking into account redundant images (Chaudhuri et al., 2018; Fan et al., 2022). However, the methodologies applied are far from the approach required to sorting with subsets restrictions and could not be integrated in the present study.

Therefore, addressing the limitation of redundancy is paramount for advancing the utility and efficiency of automated image sorting systems. Future research directions should include the development of algorithms capable of discerning and filtering out redundant images. This involves integrating more sophisticated measures that go beyond mere visual similarity, incorporating aspects such as semantic content, emotional appeal, and narrative coherence to ensure that each selected image adds distinct value to the collection. Following on the previous example of redundant images, another approach could involve leveraging image stitching algorithms to pinpoint and identify redundant photographs (Wu and Cai, 2012). Such advancements could significantly refine the process of automating commercial advertisements, offering more dynamic, engaging, and effective visual presentations.

By tackling this challenge, subsequent studies can pave the way for more intelligent automation solutions that are not only adept at organizing images but also at curating content that resonates more deeply with viewers. This progression is crucial for leveraging the full potential of automated systems in enhancing the strategic presentation of images in commercial contexts, ultimately driving greater engagement and conversion in digital marketing campaigns.

Furthermore, our study opens up several additional avenues for future research. One immediate area of interest is the exploration of different semantic features and their impact on sorting efficacy. As we have seen, certain features contribute more significantly to performance than others. A deeper understanding of these dynamics could lead to more refined algorithms that are both more efficient and effective.

Another potential direction is the investigation of alternative algorithms or enhancements to the genetic algorithm used in our study. While our approach has demonstrated promising results, there is always room for improvement in computational efficiency or sorting accuracy. Exploring machine learning techniques that can learn optimal sorting strategies from data, rather than relying on predefined semantic features, might offer new insights and improvements.

Lastly, the practical implications of our research for real-world applications should not be overlooked. Integrating our sorting solution into online platforms could significantly enhance user experience by providing more intuitive and visually coherent image arrangements. However, achieving this integration poses its own set of challenges, including scalability, platform compatibility, and user interface design considerations.

In conclusion, while our study specifically addresses the challenge of sorting images in property listings, the implications of our work extend far beyond this context. The principles and methodologies we have developed offer valuable insights and tools for a wide range of applications in online commerce and beyond. Future research, building on our findings, has the potential to further refine and expand the applications of these techniques, contributing to the advancement of the field of image processing and its practical applications.

## Data availability statement

The data analyzed in this study is subject to the following licenses/restrictions: portion of the data available upon reasonable requests sent to the authors. Requests to access these datasets should be directed to jj@udc.es.

## Author contributions

GG: Formal analysis, Methodology, Writing – original draft. MC: Methodology, Supervision, Visualization, Writing – review & editing. AT-P: Data curation, Writing – review & editing. JR: Conceptualization, Funding acquisition, Supervision, Writing – review & editing, Investigation.
